# Surgical management of sellar arachnoid cyst: state of the art and systematic review

**DOI:** 10.3389/fneur.2025.1681774

**Published:** 2025-12-18

**Authors:** Michele Pio Fabrizio, Gianpaolo Jannelli, Francesco Calvanese, Alberto Delaidelli, Andrea Cardia, Shahan Momjian, Romain Manet, Emmanuel Jouanneau, Davide Milani

**Affiliations:** 1 Department of Neurosurgery, Neurocenter of Southern Switzerland, EOC, Lugano, Switzerland; 2 Department of Neurosurgery, Kantonsspital Winterthur, Winterthur, Switzerland; 3 Department of Neurosurgery, Geneva University Hospitals, University of Geneva Faculty of Medicine, Geneva, Switzerland; 4 Department of Spinal and Robotic Surgery, Humanitas San Pio X Hospital, Milan, Italy; 5 Department of Pathology, Mass General Brigham, Harvard Medical School, Boston, MA, United States; 6 Department of Molecular Oncology, British Columbia Cancer Research Centre, Vancouver, BC, Canada; 7 Department of Cranial Neurosurgery, Pierre Weithermier Neurological Hospital, Bron, France; 8 Hôpital Neurologique et Neurochirurgical Pierre Wertheimer, Lyon, France

**Keywords:** arachnoid, cysts, sellar, intrasellar, CSF, endoscopic, transsphenoidal surgery

## Abstract

**Introduction:**

Sellar arachnoid cysts are rare intracranial lesions with variable clinical presentations, making their optimal management uncertain. This systematic review consolidates current knowledge on their epidemiology, radiological features, surgical management, and outcomes.

**Materials and methods:**

A literature search, following PRISMA-P 2015 guidelines, was conducted in MEDLINE/PubMed, Google Scholar, and Ovid Embase. Studies published in English from the year 2000 onwards were included. Data extraction focused on patient demographics, clinical presentation, surgical approaches, outcomes, and complications.

**Results:**

Thirty-three studies (16 case reports, 17 case series) met the inclusion criteria, encompassing 154 patients (59.34% female, mean age 51.48 years). The most common symptoms were visual disturbances (57.14%), headaches (35.06%), and endocrine disorders (30.52%). Surgical intervention details were available for 144 patients. Endoscopic transsphenoidal surgery was the most frequent approach (73.38%), followed by microscopic transsphenoidal surgery (11.69%). Various sellar reconstruction techniques were employed, including fascia lata, abdominal fat grafts, and nasoseptal flaps. The mean follow-up was 42.90 months. Postoperative complications occurred in 15.58% of cases, with cerebrospinal fluid leaks (7.14%) being the most common. Cyst recurrence was observed in 6.49% of patients. Most individuals with visual disturbances and headaches improved postoperatively, while endocrine function recovery was less consistent.

**Discussion and conclusions:**

SACs can cause significant morbidity due to mass effect and endocrine dysfunction. Endoscopic transsphenoidal surgery is the preferred treatment, but effective reconstruction is crucial to minimizing cerebrospinal fluid leaks. The recurrence rate highlights the importance of long-term follow-up. Future research should aim to standardize management protocols for improved outcomes.

## Introduction

Arachnoid cysts (ACs) are collections of cerebrospinal fluid (CSF) developing within the arachnoid layer of the meninges and representing approximately 1% of all intracranial mass lesions. They are typically located in the middle cranial fossa, cerebellopontine angle, quadrigeminal cistern, sellar region and vermian area (1–8). Sellar arachnoid cysts (SACs) are rare, accounting for only 3% of all intracranial ACs (1, 3–8). SACs can present with symptoms and signs of supra-parasellar mass effect and pituitary dysfunction (1, 3–5, 9). Optimal management of SACs is still debated. This is explained by their rarity as well by technical aspects of surgery which makes SACs particularly challenging among pituitary pathologies. When surgery is required, the fenestration of the cyst represents the gold standard, with several approaches and techniques described over the last two decades (1, 4, 9, 10). While both endonasal and microsurgical transsphenoidal remain the most utilized approaches, similar outcomes are achieved with transventricular or transcranial access. Regardless of the surgical technique chosen, postoperative CSF leakage have been documented with a rate higher than 20%, and both the timing and the modality of sellar reconstruction are still unclear (1, 3, 4, 6, 7, 11, 12). Finally, SACs are encumbered by a not negligible rate of recurrence, with a number of different techniques described to manage them. Current literature reports only isolated case reports or small case series and a structured systematic review on the management of SACs has not been performed yet. Our aim was to provide a systematic review exploring the relevant literature on the topic. We specifically focused on technical aspects as well as on the management of complications and recurrences.

## Materials and methods

### Search strategy and data collection

A comprehensive and systematic search was conducted in PubMed/MedLine, Google Scholar and Ovid Embase electronic databases using the keywords ‘Arachnoid’, ‘Sellar’, and ‘Cyst’ on November 13, 2023. A subsequent search was performed using the terms ‘Intrasellar’, ‘Arachnoid’, and ‘Cyst’ on November 30, 2023, in strict adherence to established scientific methodologies. Keywords were selected via a review of the literature, Medial Subject headings and Excerpta Medica Tree term. The inclusion criteria were rigorously defined and included only: studies with full-text and data availability; studies written in English; studies published after the year 2000. Due to the rarity of the pathology, case reports and small series were also included, while previous narrative series were not considered. In instances of discrepancy, the senior author (DM) acted as an arbitrator, ensuring that a consensus was reached among all authors, thus upholding the integrity of the review process. Full-text copies of all relevant articles were then retrieved, and a second search was performed from the bibliography of the selected studies. Only SACs requiring surgical treatment were included in the review. Data extraction followed a pre-defined protocol that strictly adhered to the PRISMA-P 2015 guidelines (13). The extracted data included: study characteristics (author, year, type of study); patient demographics; sample size; mean maximal lesion dimension; lesion location; symptoms; endocrinological assessments; post-operative clinical and endocrinological outcomes; surgical approach, extent of resection, and methods for sellar reconstruction; morbidity and complication rates; mortality rates; recurrence; and months of follow-up. No statistical analysis was performed due to the high heterogeneity of the data and the consequent risk of bias. This systematic review was prospectively registered in the International Prospective Register of Systematic Reviews (PROSPERO) under the registration number CRD420251006335.

## Results

### Literature review

**Figure 1:**
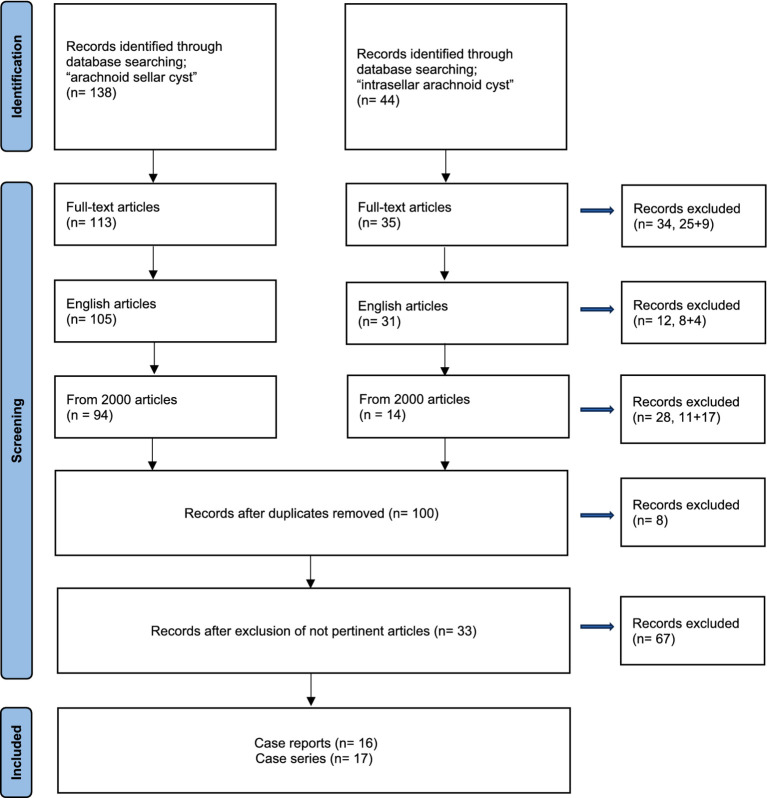
PRISMA flow-chart.

The search yielded a total of 182 references. Following the initial screening process, 100 papers were initially retained for full-text review. Of these, 67 were excluded based on relevance to the research question, leaving 33 articles for in-depth analysis. These 33 articles were composed of 16 case reports and 17 case series. The PRISMA flow-chart has been reported in Figure 1.

### Patients cohort and baseline data

A total of 33 articles were included in our analysis, consisting of 16 case reports and 17 case series, and involving 154 patients. Sex-related information was available for 91 patients. Among these, 59.34% were female (*N* = 54) and 40.66% were male (*N* = 37). Age-related information was available for 90 patients, with an average age of 51.48 years (range 6–84). Among 154 SACs, *N* = 37 (24.03%) had a suprasellar extension, while in a case the cyst extended into the middle fossa. The mean maximum diameter of SACs was available for *N* = 75 patients, with a mean value of 26.46 mm (range 8–52.6 mm). Clinically, *N* = 88 patients presented with visual disturbances (57.14%), *N* = 54 with headache (35.06%) and *N* = 47 with endocrine disorders (30.52%). In only three cases, the diagnosis was incidental.

Patient Demographics and Clinical Characteristics are reported in Table 1.

**Table 1:** Patient demographics and clinical characteristics.

Parameter	Value
Gender	Female: 54
	Male: 37
	Not stated: 63
Age (years)	Mean: 51.48
	Range: 6–84
Extension	Suprasellar: 37
	Middle fossa: 1
Maximum diameter (mm)	Mean: 26.46
	Range: 8–52.6
Symptoms (N of patiens)	Visual disturbances: 88
	Headache: 54
	Endocrine disorders: 47
	Asymptomatic: 3

### Surgical techniques

Date regarding the surgical technique were available for *N* = 144 patients and are reported in Table 2.

**Table 2:** Treatment, follow-up and outcomes.

Parameter	Category	Value (N or measure)
Surgical approaches, N		Endoscopic transsphenoidal endonasal: 113
		Microscopic transsphenoidal endonasal: 18
		Transventricular neuroendoscopic: 8
		Orbitofrontal craniotomy: 2
		Sterotactic intracavity irradiation: 4
		Not stated: 10
Follow-up, months		Mean: 42.90
		Range: 0–324
	Visual disfunction, N	Missing data: 34
		Improved: 51
		Unchanged: 1
		Worsened: 2
Clinical Outcomes	Headache, N	Missing data: 29
		Improved: 25
		Unchanged: 0
		Worsened: 0
	Endocrine disorders, N	Missing data: 32
		Improved: 10
		Unchanged: 5
		Worsened: 0
Complications, N		CSF leakage: 11
		Meningitis: 2
		Abscess: 2
		Endocrine disorders: 9
Overall complication weighted mean		29.9% (+/− 29.9 SD)
Recurrence, *N* (%)		10 (6.49)
Recurrence weighted mean		11.2% (+/− 17,8 SD)
Motality rate, %		0

Transsphenoidal endoscopic endonasal approach was performed in *N* = 113 cases (78.47%) while a microscopic TS approach was adopted in *N* = 18 cases (12.5%) (2–11, 14–29). Transcranial cyst fenestration was performed either by a neuroendoscopic transventricular approach (*N* = 8, 5.5%) or by an orbitofrontal craniotomy (*N* = 2, 1.38%) (1, 3, 12, 30, 31). One patient underwent an initial endonasal endoscopic procedure and, following a recurrence of the disease 1 year later, was treated with an endoscopic transventricular approach (3). However, less common techniques such as stereotactic intracavitary irradiation via the coronal suture using colloidal chromic-32 phosphorus (32P) were also reported (N = 4, 2.7%) (32).

### Surgical and clinical outcomes

The mean follow-up was 42.90 months (range 0–324 months). Ten cases (6.49%) presented a cyst recurrence. Eleven (7.14%) cases of CSF leakage were reported, requiring a surgical repair in seven cases. Two cases of meningitis (1.30%) and two cases of abscess (1.30%) were also reported. Regarding visual outcomes, even if a visual disorder was reported in *N* = 88 patients (57.14%), postoperative data were available for *N* = 54 cases. Among these, *N* = 51 (94%) improved, one was stable and two worsened. Similarly, postoperative data concerning headache were unavailable for 29 patients out of *N* = 54 presenting with this symptom (35.06%). All assessed patients presented an improvement in preoperative headache.

Finally, regarding endocrine disorders, a full description of both pre and postoperative symptoms was provided for only *N* = 15 patients. Among these, *N* = 10 (67%) improved and *N* = 5 remained unchanged. On the other hand, 9 (5,84%) patients with no documented preoperative endocrine dysfunction experienced a deterioration in pituitary function following surgical treatment. Of them, 3 cases presented a transient and 1 case a permanent (follow-up 11 year) syndrome of inappropriate antidiuretic hormone secretion (SIADH), four cases experienced permanent diabetes insipidus, and only one case hypocortisolism. Surgical and clinical outcomes are summarized in Table 2.

## Discussion

### Epidemiological, clinical and radiological findings

SACs are rare, accounting for approximately 1% of all intracranial cystic lesions and about 3% of all intracranial ACs (4–6). This low prevalence underscores the diagnostic challenges and the necessity for heightened clinical awareness. According to our systematic review, the mean age at presentation falls within the fifth decade of life (mean 51.48 years), with a slight female predominance (59%). Most patients present with symptoms related to mass effect, such as visual disturbances (57%) and headaches (35%) while endocrine dysfunction, though less frequent (30.5%), remains a significant clinical concern. Although only 24% of cysts exhibited suprasellar extension, visual disturbances were reported in a higher percentage of cases. This apparent discrepancy is difficult to explain. However, one possible hypothesis is that some cysts, although classified as sellar because they are located beneath the diaphragm sellae, may have exerted compressive effects on the optic pathways through stretching or deformation of the diaphragm itself under chronic pressure. This could also explain the relatively low incidence of headache, as chronic deformation of the diaphragm may, in most cases, prevent intrasellar hypertension, which is typically associated with headache. Nevertheless, this remains a speculative interpretation.

SACs should also be distinguished from suprasellar arachnoid cysts, those are typically diverticula from Liliequist’s membrane and present in children with precocious puberty, gelastic seizures, and hydrocephalus (33).

The data are not conclusive regarding SACs diagnosed incidentally. Indeed, only a few studies mention incidental diagnoses. D’Artigues et al. reported one incidental diagnosis among 17 cases, where the SAC was initially identified in the context of transient global amnesia, although subsequent ophthalmologic evaluation revealed a visual field defect (16). Somma et al. described a retrospective analysis of 78 surgical cases of rare sellar lesions, which included 12 SACs alongside a wide range of other pathologies. Among these, 14 cases were reported as incidentalomas, although the specific clinical presentation promoting radiological investigation were not specified (24). Finally, Dubuisson et al. reported other two incidental findings among nine cases of SACs (18).

Magnetic resonance imaging (MRI) remains the gold standard for diagnosing SACs. These lesions typically appear as well-circumscribed cystic masses within the sella, with signal characteristics similar to CSF on both T1- and T2-weighted images, typically with FLAIR signal suppression, without ADC restriction and non-contrast enhancing (27, 34). These characteristics help to distinguish SACs from other conditions such Rathke’s cleft cysts (variable T1 signal, intracystic nodule in 20–40% cases, non-flair suppression), cystic pituitary adenomas (enhancing solid component, fluid–fluid level, cavernous sinus invasion), craniopharyngiomas (calcifications, mixes solid and cystic parts, possible cyst T1 hyperintensity, enhancing solid components), epidermoid cysts (not full FLAIR suppression, diffusion restriction) dermoid cysts (T1 hyper intensity, fat saturation sequences suppression) and hypothalamic/chiasmatic gliomas (usually solid, contrast-enhancing masses). Additionally, SACs do not communicate with the subarachnoid space, distinguishing them from other conditions such as empty sella syndrome (27, 34).

### Pathophysiology

SACs formation is likely multifactorial, involving a combination of congenital defects, acquired pathologies, and dynamic CSF flow mechanisms. A first theory suggests that SACs develop due to herniation of the basal arachnoid membrane through a defect in the diaphragm sellae (1, 5, 6, 18). This anomaly promotes a CSF flow in the sellar region, leading to the formation of the cyst. Meyer et al. argued that SACs may arise from a splitting of the arachnoid layer, due to congenital malformation or a trauma. In this scenario, the SAC would be encapsulated within arachnoid membranes, without any communication with the subarachnoid space (35). According to “the ball-valve” mechanism, a small defect in the diaphragm promotes the inflow of CSF in the sellar region during periods of increased intracranial pressure while the outflow is restricted by surrounding structures, as the pituitary gland (10, 18, 36). The CSF flow entering the cyst but not returning has been also observed intraoperatively, supporting the validity of this one-way valve mechanism (10). Finally, another hypothesis supports the theory that SACs arise from adhesions or scarring within the arachnoid membranes, triggered by previous infections (e.g., meningitis), hemorrhage, or trauma. These adhesions can impair physiological CSF flow and promote cyst formation (18).

### Management

#### Surgical treatment

Surgical intervention is generally required for symptomatic SACs presenting with visual and/or endocrine disturbances. In our systematic review, TS endonasal approach was performed in 73.38% of cases (Tables 3–5). This approach offers several advantages, including superior visualization and reduced morbidity (15). Transcranial routes, such as the neuroendoscopic transventricular approach or microsurgical fenestration via orbitofrontal or frontolateral craniotomy, have been employed in cases with significant suprasellar extension or when transsphenoidal access is challenging for anatomical reasons (31) (Table 6). Shim et al. implemented a transventricular endoscopic fenestration for six patients with significant suprasellar cyst extension (31). This method avoids complications such as CSF leaks present in transsphenoidal surgery, by creating a direct connection of cystic compartment with subarachnoid space, reducing recurrence risks without requiring shunting. Navigation-assisted neuroendoscopic approach allow access to the third ventricle through a simple burr hole and allow suprasellar decompression without pituitary manipulation. Although further studies on long-term outcomes are warranted, this approach led to a substantial reduction in cyst volume and symptom relief in all cases, with minimal complications, as reported in the series by Shim et al. (31). The surgical risks are similar to those of a standard third ventriculostomy, including potential injury to the hypothalamus, visual pathways (due to proximity to the optic tract and chiasm), and vascular structures. In our systematic review, we collected outcome data on 70 patients treated with the transnasal-transsphenoidal approach and 8 patients treated with the transventricular approach. Among those treated with the transnasal-transsphenoidal approach, of the 23 patients presenting with headaches, 20 reported symptom improvement (87%); of the 49 patients with visual disturbances, 42 experienced clinical improvement (86%); and of the 14 patients with hormonal disturbances, 6 showed clinical and laboratory improvements (43%). Among those treated with the transventricular approach: of the 2 patients with headaches, both reported symptom improvement; of the 8 patients with visual disturbances, 7 demonstrated clinical improvement (87.5%); and of the 4 patients with hormonal disturbances, 3 achieved clinical and laboratory improvement (75%).

**Table 3:** Articles on endoscopic endonasal approach with their strategies, outcomes, complications and follow-up (part 1).

Year, author	Sample size (n)	Gender (*n*, M/F)	Mean age (years +/− SD, range)	Symptoms and outcome	Endocrine Assessment and Outcome	Location	Mean maximal diameter (mm +/− SD, range)	Surgical approach and recostruction	Extent resection	Morbidity rate/complications (%)	Mortality rate (%)	Recurrence (% during FU)	Follow up (months)
Yasuda et al. (29)	1	M	67	Visual field defect; Endocrine disturbance; Headache Post-op headache improvement; No visual field defect or endocrine disturbance improvement	Hypothyroidism Hypocortisolism; No post-op improvement	Sellar	40 mm	Endonasal transsphenoidal approach; recostruction with cartilage, fibrin glue and fatty tissue	Fenestration and partial membrane resection	0%	0%	0%	6
Cavallo et al. (15)	10	/	/	Headache (20%) Visual field defect (70%) Impotence (30%) Hyperprolactinemia (20%) Post-op headache improvement (50%), visual defect improvement (100%), impotence improvement (0%), Hyperprolactinemia improvement (100%)	Impotence (30%) Hyperprolactinemia (20%) Post-op impotence improvement (0%), Hyperprolactinemia improvement (100%)	Sellar	/	Endoscopic endonasal approach; recostruction with sellar packing (fat graft and/or collagen sponge) and sellar floor reconstruction	Fenestration	2 CSF fistula (20%) and 1 with meningitis (10%)	0%	1 recurrence after 16 months (10%) Treated with reoperation	36.9 (10–94)
McLaughlin et al. (6)	8	2 M 6\u00B0F	57 (43–81)	Headache (50%) Pituitary dysfuntion (50%) Visual dysfuction (50%) Resolution after surgery of all symptoms and partial resolution of endocrine dysfunction	Hypogonadism (50%) Hyperprolactinemia (25%) Hypoadrenalism (37.5%) Hypothyroidism (25%) Recurrent Hyponatremia (25%) Just little improvement post-op	Sellar	22 mm (range 15–32 mm)	Endoscopic endonasal transsphenoidal fenestration and obliteration with fat graft	No resection > fenestration and obliteration	0%	0%	2/8 (25%) > one of this was reoperated	Clinical mean 32 (range 10–53); Imagin mean 21 (6–47)
Barkhoudarian et al. (11)	8	/	/	Headache, pitiutary disfunction and vision loss	/	/	/	Endoscopic endonasal transsphenoidal approach	/	/	/	/	/
Bordo et al. (14)	8	only data for 3 patients with Hyponatremia (1 M 2 F)	only data for 3 patients with Hyponatremia (74.3, range 67–84)	Hyponatremia (37.5% of all 8 patients) Only data for 3 patients with Hyponatremia (Hypogonadism 100%, Hypoadrenalism 100%, IGF-1 deficiency 66.6%, Hypothyroidism 66.6%)	Hyponatremia (37.5% of all 8 patients) Only data for 3 patients with Hyponatremia (Hypogonadism 100%, Hypoadrenalism 100%, IGF-1 deficiency 66.6%, Hypothyroidism 66.6%)	Sellar	35 mm	Endoscopic endonasal transsphenoidal	/	0%	0%	0%	14 (2–46)
Dawkins et al. (17)	1	M	67	Visual defect Post-op improvement	Normal	Sellar with suprasellar extension	/	Endoscopic endonasal transsphenoidal; recostruction with abdominal graft, nasoseptal flap	Fenestration and partial membrane resection	0%	0%	0%	6
Su et al. (25)	3	1 M 2 F	51 (37–64)	Visual disturbance (100%) Headache (33%)	Normal	Sellar	39 (range 27–52 mm)	Endoscopic transsphenoidal surgery with intentional fenestretione to the subarachnoid space and closing the sellar floor using delicate durale suturing technique (no fat use)	No resection, only fenestration and suture	0%	0%	/	6
Güdük et al. (2)	1	M	49	Headache	Normal	Sellar and suprasellar	18 mm	Endoscopic endonasal transsphenoidal approach	Fenestration and total membrane resection	0%	0%	0%	12
Somma et al. (24)	12	/	/	Headache, pitiutary disfunction and vision loss	/	Sellar (91%) and suprasellar (66%)	/	Endoscopic endonasal transsphenoidal approach	Total Removal 100%	/	/	/	/
Castle-Kirszbaum et al. (10)	1	F	51	Visual disturbance and defect; Headache Post-op haedache and visual fields improvement	Normal	Sellar	27 mm	Endoscopic endonasal approach; recostruction with fat graft and nasoseptal flap	Fenestration	SIADH	0%	0%	/
Chen et al. (15)	1	F	35	Visual field defect; Post-op improvement	Normal	Sellar	/	Endoscopic endonasal transsphenoidal approach; recostruction with artificial dura mater, gelatin sponge, fatty tissue and mucosal flap repairment	Fenestration	CSF post-operative leak treated ESD; persistence of CSF leak treated with reoperation	0%	0%	/
Roca et al. (22)	10	/	/	/	/	Sellar	/	Endoscopic endonasal transsphenoidal approach; recostruction with abdominal fat graft	/	2 CSF fistula (20%) with reoperation	0%	/	/

**Table 4:** Articles on endoscopic endonasal approach with their strategies, outcomes, complications and follow-up (part 2).

Year, author	Sample size (n)	Gender (n, M/F)	Mean age (years +/− SD, range)	Symptoms and outcome	Endocrine Assessment and Outcome	Location	Mean maximal diameter (mm +/− SD, range)	Surgical approach and recostruction	Extent resection	Morbidity rate/complications (%)	Mortality rate (%)	Recurrence (% during FU)	Follow Up (months)
Mangussi-Gomes et al. (19)	1	F	36	Headache; Visual defect; Endocrine disturbance; No post-op informations	Hyperprolactinemia; Hypocortisolemia; reduced IGH No post-op informations	Sellar with suprasellar extension	/	Endoscopic endonasal approach	Decompression (fenestration?)	0%	/	/	
Ovenden et al. (21)	1	F	45	Bitemporal hemianopia	/	Sellar with suprasellar extension	29 mm	Endoscopic endonasal transsphenoidal approach; recostruction with Duragen and Durasis	Fenestration only	Pituitary ascess > treatment with antibiotics and second surgery	0%	0%	6
Tafreshi et al. (26)	2	/	/	Vision loss and headache (100%) Resolution after surgery	/	Sellar (1 with suprasellar extension)	/	Endoscopic endonasal approach > Fenestration	No resection, only fenestration	Post-operative CSF leak (50%)	0%	/	/
d’Artigues et al. (16)	17	5 M 12\u00B0F	46.6 (17–82)	Headache (47%) Visual disturbance (64.7%) Pituitary disfunction (17.6%) Post-op visual improvement (83.3%), visual worsening (11.7%), headache improvement (87.5%)	Hypothyroidism (11.7%) Hypogonadism (5.8%) Post-op > no improvement	Sellar	24 mm (range 16–42 mm)	Endoscopic endonasal transsphenoidal approach; only fenestration, fat graft and Titanium Mesh (to maintain the fat in its position)	No resection, only fenestretion	CSF post-operative leak (11.8%); Permanent diabetes insipidus (11.8%); Temporary sever hyponatremia (SIADH) (5.8%)	0%	1 recurrence at 3 months (5.8%)	39 (3–106)
Tavakol et al. (27)	15	/	/	/	/	Sellar	/	Endoscopic transsphenoidal approach	/	/	/	/	/
Jannelli et al. (3)	1	/	77	Visual disturbance > Endoscopic endonasal approach (without fat) Post-op CSF rhinorrhea > reoperation with fat graft 1 year recurrence with visual disturbance > endoscopic transventricular approach to put Sensor Reservoir	Normal	Sellar	/	(1) Endoscopic endonasal approach (without fat) (2) reoperation and fat graf application (3) endoscopic transventricular approach to put Sensor Reservoir	Fenestration; Drainage	CSF post-operative leak	0%	Recurrence after second surgery	24
Kalyvas et al. (4)	10 (8 new diagnosys; 2 recurrence)	3 M 7\u00B0F	54.5 (20–77)	Headache (10%) Visual symptoms (90%) Hypopitituarism (20%) Post-op visual improvement (60%)	Hypopitituarism (20%) Post-op improved	Sellar	17 mm (range 8–28 mm)	Endoscopic endonasal transsphenoidal approach; only fenestration, dura substitute, and nasoseptal flap (no fat use)	No resection, only fenestration and small biopsy	New hypocortisolism (10%); New diabetes insipidus (10%)	0%	1 recurrence at 54 months (10%); 2 recurrence in selected group (20%)	85.5 (11–158)
Matmusaev et al. (5)	1	F	74	Bitemporal hemianopia and diminished visual acuty	Normal	Sellar	30 mm	Endoscopic endonasal transsphenoidal approach	No resection > fenestration and obliteration	0%	/	/	24
Silva et al. (23)	1	F	51	Visual field defect	Normal	Sellar with left middle fossa extension	/	Endoscopic endonasal approach; recostruction with cellulose, fibrin glue, abdominal fat and fascia, nasal cartilage, nasoseptal flap	Fenestration	/	/	/	/

**Table 5:** Articles on microscopic endonasal approach with their strategies, outcomes, complications and follow-up.

Year, author	Sample size (n)	Gender (n, M/F)	Mean age (years +/− SD, range)	Symptoms and outcome	Endocrine Assessment and Outcome	Location	Mean maximal diameter (mm +/− SD, range)	Surgical approach and recostruction	Extent resection	Morbidity rate/complications (%)	Mortality rate (%)	Recurrence (% during FU)	Follow Up (months)
Weil (28)	1	F	74	Headache Visual field defect Post-op headache and visual field defect improvement	Normal	Sellar and suprasellar	/	Microscopic sublabial transseptal transnasal approach; reconstruction with fat graft and nasoseptal bone	Resection	0%	0%	0%	6
Murakami et al. (20)	1	M	48	Visual field defect Post-op visual field defect improvement	Normal	Sellar	/	Microscopic transsphenoidal approach; reconstruction with fat graft and bony septum	Fenestration	0%	0%	After 4 years; second surgery with right Sylvian approach	48
Dubuisson et al. (18)	9	5 M 4\u00B0F	45 (20–80)	Headache (22.2%) Vision disturance (33%) Post-op visual improvement (100%), headache improvement (50%)	Panhypituitarism (11.1%) Hyperprolactinemia (33.3%) Hypogonadism (44.4%) Post-op partial hypopituitarsm improvement (80%), hyperprolactinemia normalization (100%).	Sellar and suprasellar (66.6%)	24 mm (range 12–35 mm)	Microscopic transsphenoidal approach; recostruction with adipose tissue, nasoseptal bone, biological glue.	Fenestration and total membrane resection	CSF post-operative leak (22.2%) with reoperation; Permanent diabetes insipidus (11.1%)	0%	0%	132 (2–324)
Park et al. (8)	1	F	53	Headache Diabetes insipidus No post-op diabetes insipidus change	Mildly eleveted prolactin No post-op change	Sellar	9 mm	Microscopic transsphenoidal approach; recostruction with gelfoam, fat tissue and nasoseptal bone	Cyst removal > cyst fluid appear infected	/	0%	0%	12
Oyama et al. (7)	6	3 M 3\u00B0F	59 (range 55–65)	Visual field defect (100%) Post-op visual field defect improvement (100%)	Normal	Sellar and suprasellar (100%)	37 mm (range 26–37 mm)	Microscopic transsphenoidal approach and endoscopic inspection; reconstruction with fascia lata, fat graft, dural 6–0 suture	Fenestration and cisternostomy with a keyhole	1 CSF leak and meningitidis treated by lumbar drainage and antibiotics (16.6%)	0%	1 recurrence (16.6%)	42.2 (range 36–49)

**Table 6:** Articles on craniotomy approach with their strategies, outcomes, complications and follow-up.

Year, author	Sample size (n)	Gender (n, M/F)	Mean age (years +/− SD, range)	Symptoms and outcome	Endocrine Assessment and Outcome	Location	Mean maximal diameter (mm +/− SD, range)	Surgical approach and recostruction	Extent resection	Morbidity rate/complications (%)	Mortality rate (%)	Recurrence (% during FU)	Follow Up (months)
Shim et al. (31)	6	2 M 4\u00B0F	45 (range 27–47)	Visual defect (100%); Endocrine disturbance (66.6%); Headache (33.3%); Post-op visual defect improvement (83.3%); endocrine disturbance improvement (75%); no information about headache	Hyperprolactinemia (16.6%); Diabetes insipidus (16.6%); Hypogonadism (33.2%); Post-op endocrinological improvement, except for DI	Sellar and suprasellar	35.5 mm (range 25.3–52.6)	Transventricular endoscopic approach with fenestration	Fenestration and partial membrane resection	Transient SIADH (33.3%)	0%	0%	10 (range 6–12)
Edvardsson and Persson (30)	1	M	43	Headache Post-op improvement	Normal	Sellar with suprasellar extension	/	Craniotomy with cyst fenestration	Fenestration and partial membrane resection	0%	0%	0%	4
Aljuboori et al. (1)	1	F	64	Visual disturbance and defect; Headache; Diabetes insipidus Post-op headache and visual defect improvement; DI had resolved	Diabetes insipidus Post-op improvement	Sellar	9.4 mm	Orbitofrontal craniotomy	Fenestration	Palpebral abscess	0%	0%	6
Sasaki et al. (12)	1	M	82	Visual defect Post-op improvement	Hypothyroidism Hypocortisolism; Post-op partial improvement	Sellar	20 mm	Transventricular endoscopic fenestration	Fenestration and partial membrane resection	0%	0%	0%	24
Jannelli et al. (3)	1	/	77	Visual disturbance > Endoscopic endonasal approach (without fat) Post-op CSF rhinorrhea > reoperation with fat graft 1 year recurrence with visual disturbance > endoscopic transventricular approach to put Sensor Reservoir	Normal	Sellar	/	(1) Endoscopic endonasal approach (without fat) (2) reoperation and fat graf application (3) endoscopic transventricular approach to put Sensor Reservoir	Fenestration; Drainage	CSF post-operative leak	0%	Recurrence after second surgery	24

With the improvement of endoscopic techniques, the literature increasingly supports the transsphenoidal approach as the firstline option. Transcranial or neuroendoscopic techniques may be preferred when anatomical contraindications to the inferior approach are present.

#### Complete cyst removal or fenestration?

Complete cyst resection was reported rarely, instead, fenestration and drainage were the primary strategy to alleviate mass effect (1). In our systematic review, we noted that most authors prefer not to perform complete (reported in only 16.6% of cases) or partial dissection of the cyst walls, as similar decompressive effectiveness can be achieved with drainage or fenestration, while reducing the risk of endocrinological complications and CSF leakage.

Dubuisson et al. treated a series of 9 SAC patients with drainage and partial (7/9) or complete excision by microscopic transsphenoidal approach (18). For cysts communicating with the subarachnoid space, a broad fenestration toward the suprasellar cisterns ensured effective drainage. In these cases, the authors highlighted the importance of meticulous reconstruction of the inferior cyst wall and sellar floor using fat packing, nasal bone, and biological glue, and indicated that the addition of postoperative lumbar drainage further contributed to reducing the risk of CSF leakage. They obtained an improvement of the preoperative visual deficit in all three patients and a normalization of the prolactin levels in all cases. Concerning complications, one patient developed permanent diabetes insipidus while CSF leak occurred in 2 patients, leading to reoperation when persistent.

Dubuisson et al. underscore that with meticulous closure and dissection, SAC can be partially or completed resected by transsphenoidal approach effectively relieving optic and pituitary compression and allows histological confirmation through partial or complete excision of the cyst membrane as well as reduction of the recurrence rate (18).

On the other hands, D’Artigues et al. treated 17 patients with symptomatic SACs with simple drainage by an endoscopic endonasal transsphenoidal approach (16). After draining the cystic CSF and inspecting the cavity with an endoscope to rule out tumoral components, the cyst wall was not dissected or fenestrated toward suprasellar cisterns to reduce CSF leakage risk. A waterproof closure was achieved with a fat graft harvested from subcutaneous abdominal tissue, layered in the sella, and reinforced with collagen patches and titanium mesh. Although two patients experienced postoperative CSF leaks, both cases were successfully resolved with reoperation and large grafting. The technique achieved symptom relief in most patients, with improved visual outcomes in 83.3% and headache relief in 87.5%. The authors argue that sparing the cyst wall minimizes pituitary damage and decreases CSF leakage risks, making this technique valuable in managing SACs. Similarly, Oyama et al. developed a novel cisternostomy method involving a small transsphenoidal keyhole incision in the sellar dura on a series of 7 patients (7). This approach combined a fenestration into the prechiasmatic and prepontine cisterns sparing brain tissue manipulation. Oyama’s technique minimizes CSF leak risk through secure dural repair with fascia lata and monofilament sutures. All patients reported substantial postoperative improvement, with only two cyst recurrences over a 42.2-month follow-up, both successfully managed with reoperation. In conclusion, there are no definitive data supporting or opposing resection versus simple drainage of SACs. Despite a potentially higher risk of recurrence, simple drainage should be preferred for relieving pressure on the sellar and suprasellar structures and for symptom control, as it reduces the risk of pituitary manipulation and potential CSF leakage.

#### Alternative techniques

We previously described a challenging case of a 77-year-old patient with visual field deficits due to a SAC compressing the optic chiasm (3). Initially, the cyst was treated with an endoscopic endonasal approach without fat reconstruction; however, the patient later developed a CSF leak, which required additional surgical repair. Subsequent cyst recurrence and visual worsening led to a transcranial fenestration attempt, but the cyst regrew again. As standard treatments had failed, the authors placed an intracystic catheter via a transventricular approach, connecting it to a telemetric Sensor Reservoir® that allowed both intracystic pressure monitoring and CSF drainage when pressure rose. This approach stabilized the patient’s vision, demonstrating a novel solution for complex, recurrent SAC cases when conventional methods are insufficient. Stereotactic techniques also offer less invasive options for cyst stabilization. Güzel et al. treated a 3-year-old girl with precocious puberty caused by a suprasellar cyst using stereotactic internal drainage, accessed through a frontal burr hole, which provided long-term stability while avoiding open craniotomy risks (37). This method allowed symptom resolution within a year, with sustained cyst stability and normal growth and endocrine function over a 20-year follow-up. Stereotactic intracavitary irradiation, as described by Thompson et al. in a series of four patients, achieved an average cyst volume reduction of 77.5% with no recurrences or complications by delivering a targeted dose of phosphorus-32 (^32^P) to the cyst wall (32). This technique is beneficial for patients without cyst communication with basal cisterns, minimizing tissue exposure and eliminating radiation precautions for healthcare staff and family members. Alternative techniques are described in Table 7.

**Table 7:** Articles on other or not specified approach with their strategies, outcomes, complications and follow-up.

Year, author	Sample size (n)	Gender (n, M/F)	Mean age (years +/− SD, range)	Symptoms and outcome	Endocrine Assessment and Outcome	Location	Mean maximal diameter (mm +/− SD, range)	Surgical approach and recostruction	Extent resection	Morbidity rate/complications (%)	Mortality rate (%)	Recurrence (% during FU)	Follow Up (months)
Thompson et al. (32)	4 (1 case after stereotactic aspiration of the cyst, 1 case after transsphenoidal biopsy of the cyst)	1 M 3\u00B0F	51.7 (46–73)	Headache (100%) Vision disturbance (100%) Post-procedure headache improvement (100%), visual disturbance improvement (25%)	/	Sellar and suprasellar (100%)	Maximum volume 13.9 cc (range 5.6–13.9 cc)	Stereotactic intracavitary irradiation through coronal suture using chromic colloidal phosphorus-32 (32P) - 0.8 mL	No resection > stereotactic intracavitary irradiation using chromic colloidal phosphorus-32 (32P)	0%	0%	0%	(9, 10, 15–17, 33)
Buslei et al. (40)	8	6 M 2 F	39 (6–61)	/	/	Sellar	/	/	/	/	/	/	/
Xu et al. (34)	2	2 M	Children	Short stature	Growth ormon deficiency (100%)	Sellar	/	/	/	/	/	/	/

These approaches highlight the spectrum of effective SAC management options, where minimally invasive endoscopic transsphenoidal surgery (TS) is frequently preferred for its lower morbidity and direct access.

#### Postoperative CSF leak

Post-operative CSF leak represents the principal complication, occurring in up to 17% of SAC surgery, depending on the surgical approach and the presence of intraoperative leaks (22), is critical to avoid. In our systematic review, CSF leaks occurred in 7.14% of cases. The use of abdominal fat, fascia lata, nasoseptal flap, dural sutures and fibrin glue have been suggested to be effective in reducing the incidence of postoperative CSF leaks (3, 4, 6–10, 15–18, 20, 22, 23, 25, 28, 29). For instance, Roca et al. reported a persistent CSF leak rate of 3.7% following the use of abdominal fat grafts, underscoring the effectiveness of this technique (22). In addition to its application for sellar floor reconstruction, fat grafting has also been employed within the sellar arachnoid cyst cavity itself, as an adjunctive measure to enhance sealing and minimize postoperative CSF leakage (6, 16, 18, 22). The use of autologous fat carries a theoretical risk of visual deterioration. In our review, no cases of new visual field deficits attributable to fat packing were identified in SAC surgery. However, in broader endonasal skull base practice, fat grafts have occasionally been implicated in postoperative visual decline, typically due to compressive mechanisms such as overpacking or graft migration leading to chiasmal compression (38). These complications, though uncommon, underscore the importance of cautious graft placement and postoperative vigilance, as timely recognition and surgical revision are crucial to prevent permanent visual loss.

#### Recurrence

Recurrence of SACs remains also a major concern, with a pooled rate of 6.49% in our systematic review. Strategies to manage recurrences include repeat fenestration and the use of vascularized nasoseptal flaps. In refractory cases, stereotactic intracavitary irradiation has been suggested as an effective intervention (32). Cabrilo et al. describe the use of a custom cruciform drain to create a long term stoma between the cyst lumen and the suprasellar cisterns to prevent further recurrence (39). Routine postoperative imaging and long-term follow-up are crucial for detecting early recurrences and managing them promptly. Monitoring intracystic pressure in complex multioperated cases may aid in early detection of recurrence (3), though its effectiveness needs further study. Effective SAC management requires a tailored approach that considers individual cyst characteristics and patient factors, with endoscopic and stereotactic advances expanding the range of effective, low-morbidity options for SACs.

### Proposed management algorithm

**Figure 2:**
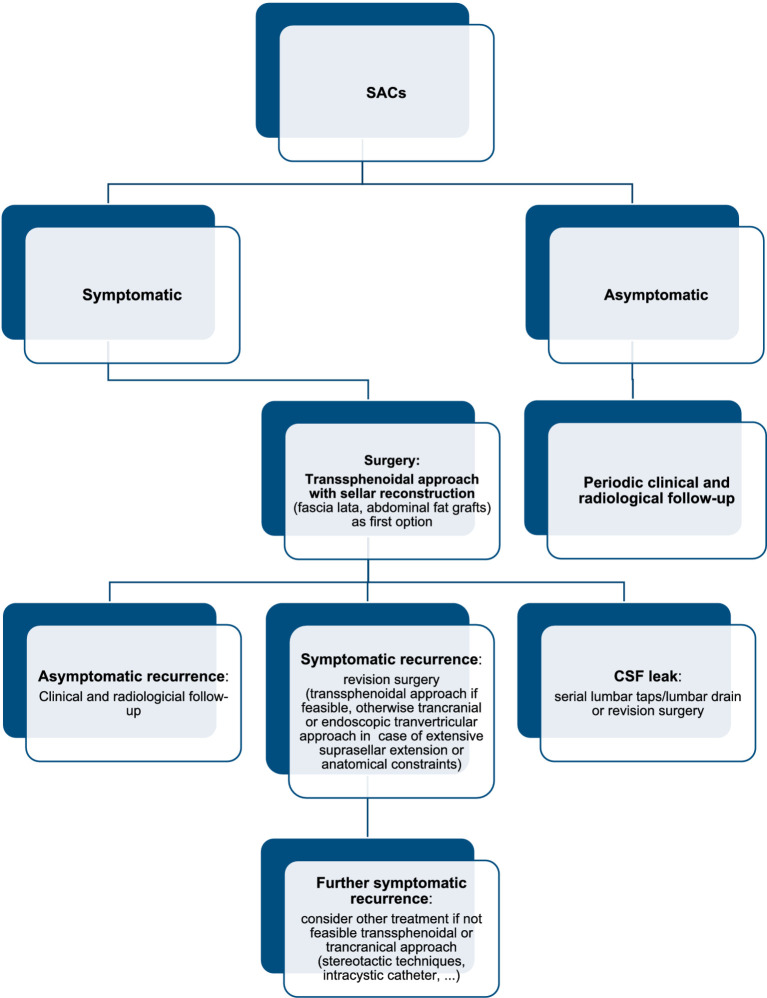
Management flowchart.

**Figure 3:**
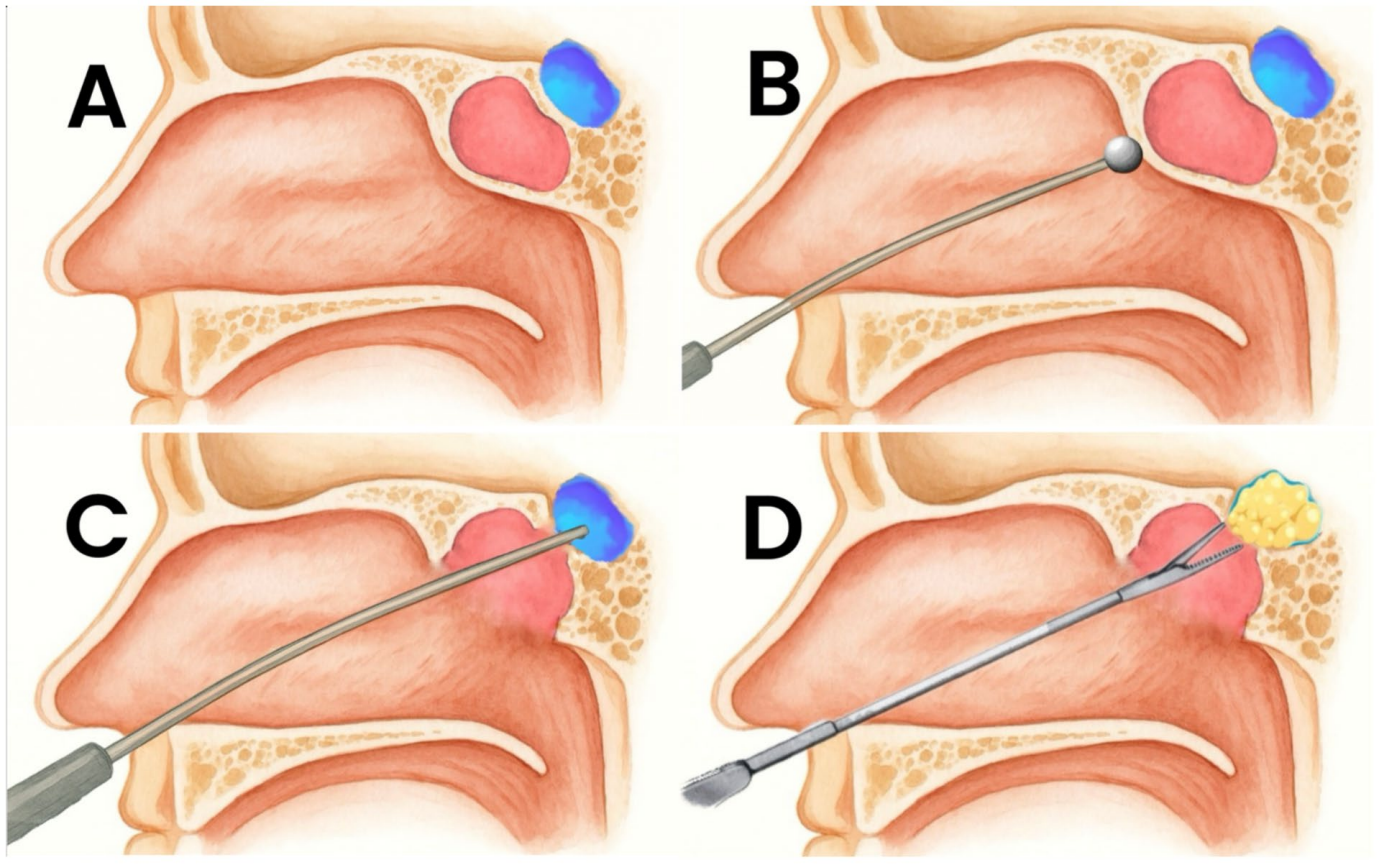
Illustrative example of the endoscopic transsphenoidal workflow for the management of a sellar arachnoid cyst: **(A)** sagittal schematic showing the sellar arachnoid cyst (blue) within the sella turcica; **(B)** endoscopic transsphenoidal access with a surgical burr used to open the sphenoid sinus and expose the anterior aspect of the cyst; **(C)** wide fenestration of the cyst, with preservation of the cyst wall; **(D)** sellar reconstruction with fat packing; the residual blue rim indicates the remaining cyst capsule after fenestration.

Basing on articles included in our review, management of SACs requires a stepwise approach based on clinical presentation, imaging findings, and surgical considerations. Asymptomatic or incidentally discovered SACs generally warrant conservative management with periodic clinical and radiological follow-up to monitor potential progression. Symptomatic cases, particularly those with visual disturbances, endocrine dysfunction, or significant mass effect, require surgical intervention. The transsphenoidal approach, preferably performed using an endoscopic technique, represents the first-line surgical option due to its minimally invasive nature, direct access to the sella, and favorable outcomes (2–11, 14–29). Similarly to the majority of sellar pathologies requiring surgical treatment, transcranial or transventricular endoscopic approach should be limited to cases with extensive suprasellar extension, anatomical constraints or cyst recurrence where transsphenoidal approach may be difficult (1, 3, 12, 30, 31). Intraoperatively, wide fenestration of the cyst wall into the subarachnoid cisterns is preferred over complete excision to reduce the risk of CSF leakage and pituitary dysfunction (1, 7, 16, 18). Due to a higher risk of CSF leak, sellar reconstruction remains a critical step. Compared to conventional transsphenoidal surgery practice, our analysis suggests utilizing autologous fat grafting and fascia lata, as for more extended transsphenoidal approaches (3, 6–10, 15–18, 20, 22, 23, 28, 29). Furthermore, placement of the fat graft not only for sellar floor reconstruction but also within the arachnoid cyst cavity itself is advised, in order to promote sealing and reduce the risk of postoperative CSF leakage (6, 16, 18, 22). Serial postoperative lumbar taps or the use of a lumbar drain may be also considered in order to further reduce the risk of CSF leak (6, 7, 15, 18). Long-term follow-up is essential, as recurrence occurs in a subset of cases, requiring repeat intervention, which may include a second fenestration or alternative techniques such as stereotactic cyst aspiration or shunting, in refractory cases (3, 4, 6, 7, 15, 16, 18, 20, 32). Flowchart is presented in Figure 2. An illustrative example of the endoscopic transsphenoidal workflow for the management of a sellar arachnoid is presented in Figure 3.

### Recommended postoperative follow-up

Given the rarity of sellar arachnoid cysts, no formal postoperative surveillance guidelines are currently available in the literature. Moreover, available data do not identify a characteristic timeframe for recurrence, and recurrence rates do not appear to differ significantly between surgical techniques. These uncertainties further underscore the need for structured and prolonged follow-up. To assist clinicians in decision-making, we propose general expert-based recommendations for postoperative monitoring. We suggest early endocrinological assessment within 2–3 weeks after surgery to identify transient or delayed hormonal disturbances. A comprehensive evaluation—including MRI, neuro-ophthalmologic examination with formal visual field testing, and endocrinological assessment—should then be performed at 3 months postoperatively and subsequently on an annual basis for at least 5 years. Beyond this period, follow-up should be individualized based on clinical symptoms and radiological stability. These recommendations apply irrespective of the surgical approach used, as no technique-specific differences in recurrence have been demonstrated.

## Conclusion

SAC, though rare, require a nuanced approach to diagnosis and management. Surgical intervention remains the mainstay of treatment for symptomatic cases, with the endoscopic transsphenoidal approach being the most widely adopted technique. Management of postoperative complications, particularly CSF leaks, and close monitoring for recurrence are crucial for ensuring optimal patient outcomes. Further research into the long-term outcomes of different surgical techniques and the role of advanced imaging in early detection of recurrences will help refine management strategies for this challenging condition.

## Data Availability

The original contributions presented in the study are included in the article/supplementary material, further inquiries can be directed to the corresponding author/s.
